# Jensen–Shannon divergence framework for quantifying gene-centric differences between matched bulk and single-cell RNA-seq breast cancer datasets

**DOI:** 10.1093/bib/bbaf631.061

**Published:** 2025-12-12

**Authors:** Md Mamunur Rashid, Kumar Selvarajoo

**Affiliations:** Bioinformatics Institute (BII), Agency for Science, Technology and Research (A*STAR), 30 Biopolis Street, Matrix #07-01, Singapore, 138761, Republic of Singapore; Bioinformatics Institute (BII), Agency for Science, Technology and Research (A*STAR), 30 Biopolis Street, Matrix #07-01, Singapore, 138761, Republic of Singapore

## Abstract

**Motivation:**

Bulk RNA sequencing (RNA-seq) captures tissue-level transcriptomes that reflect tumor-intrinsic programs and microenvironmental signals, while single-cell RNA-seq (scRNA-seq) enables analysis of cellular heterogeneity. Pseudo-bulk (PB) RNA-seq, generated by aggregating scRNA-seq profiles, has become a standard surrogate for bulk in benchmarking deconvolution, differential expression (DE), and synthetic data generation. However, it remains unclear whether PB faithfully represents true bulk transcriptomes.

**Methods:**

We introduce a gene-centric Jensen–Shannon Divergence (JSD) framework, a model-free, information-theoretic approach to quantify PB–bulk differences at single-gene resolution using matched breast cancer datasets. Genes were stratified into low-JSD ‘stable proxies’ and high-JSD ‘divergent drivers,’ followed by functional enrichment and validation in scRNA-seq clusters.

**Results and Conclusion:**

High-JSD genes (≈25–30% unique) drive systematic PB–bulk divergence and introduce spurious correlations, largely overlooked by standard DE or PCA analytics. Bulk-specific divergent genes are enriched for stromal and immune pathways, reflecting tumor microenvironmental signals. In contrast, PB-specific divergent genes highlighted cell-autonomous processes, including metabolism and transcriptional regulation in endothelial, T-cell, and myeloid cells. Low-JSD genes provide stable cross-platform signals, improving gene-level similarity, batch correction, and alignment. This framework disentangles modality-specific biases in cancer transcriptomics and identifies robust gene subsets for reliable bulk–single-cell integration.

**References:**

1. Jin W, Pei J, Roy JR et al. Comprehensive review on single-cell RNA sequencing: A new frontier in Alzheimer’s disease research, Ageing Res Rev 2024;100:102454.

2. Jew B, Alvarez M, Rahmani E et al. Accurate estimation of cell composition in bulk expression through robust integration of single-cell information, Nat Commun 2020;11:1971.

